# Towards global flood mapping onboard low cost satellites with machine learning

**DOI:** 10.1038/s41598-021-86650-z

**Published:** 2021-03-31

**Authors:** Gonzalo Mateo-Garcia, Joshua Veitch-Michaelis, Lewis Smith, Silviu Vlad Oprea, Guy Schumann, Yarin Gal, Atılım Güneş Baydin, Dietmar Backes

**Affiliations:** 1 Universidad de Valencia, Valencia, Spain; 2 Liverpool John Moores University, Liverpool, UK; 3 University of Oxford, Oxford, UK; 4 University of Edinburgh, Edinburgh, UK; 5 University of Bristol, Bristol, UK; 6 RSS-Hydro, RED, Dudelange, Luxembourg; 7 University of Luxembourg, Luxembourg, Luxembourg; 8 University College London, London, UK

**Keywords:** Environmental sciences, Hydrology, Natural hazards, Computer science, Scientific data, Software

## Abstract

Spaceborne Earth observation is a key technology for flood response, offering valuable information to decision makers on the ground. Very large constellations of small, nano satellites— ’CubeSats’ are a promising solution to reduce revisit time in disaster areas from days to hours. However, data transmission to ground receivers is limited by constraints on power and bandwidth of CubeSats. Onboard processing offers a solution to decrease the amount of data to transmit by reducing large sensor images to smaller data products. The ESA’s recent PhiSat-1 mission aims to facilitate the demonstration of this concept, providing the hardware capability to perform onboard processing by including a power-constrained machine learning accelerator and the software to run custom applications. This work demonstrates a flood segmentation algorithm that produces flood masks to be transmitted instead of the raw images, while running efficiently on the accelerator aboard the PhiSat-1. Our models are trained on *WorldFloods*: a newly compiled dataset of 119 globally verified flooding events from disaster response organizations, which we make available in a common format. We test the system on independent locations, demonstrating that it produces fast and accurate segmentation masks on the hardware accelerator, acting as a proof of concept for this approach.

## Introduction

**Figure 1:**
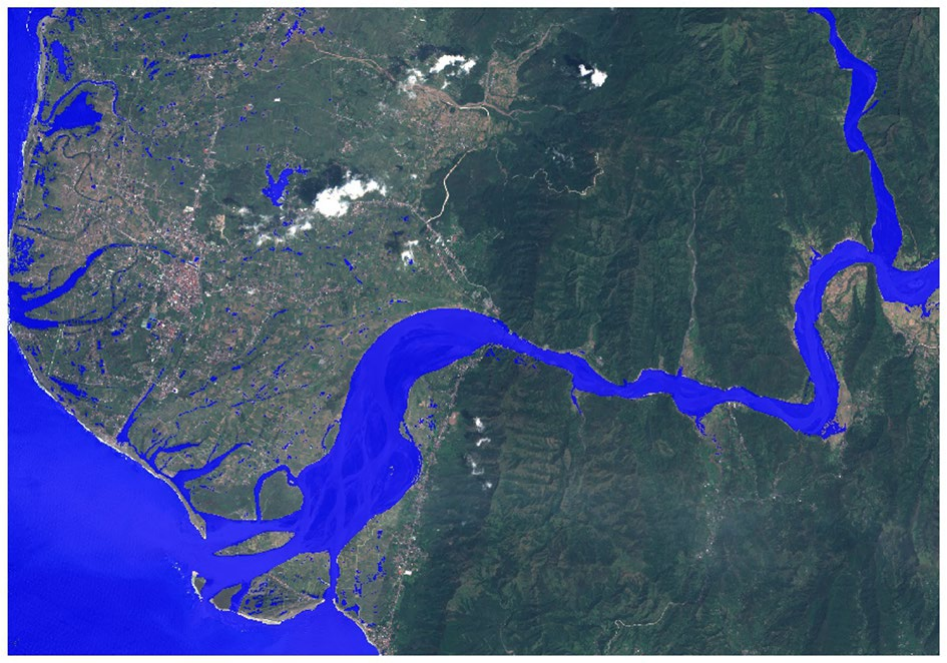
An example of a data product from the Copernicus EMS catalogue (activation EMSR312), in this case a map showing flood extent over the city of Vigan in the North West of Luzon island in the Philippines in September 2018. A blue water mask (here generated using an automatic method from a RADARSAT-2 image) is overlaid on top of a Sentinel-2 image, showing the extent of flooding. Sentinel 2 imagery and Copernicus EMS mapping products are provided as public domain. Base image and reference labels mask are included in the *WorldFloods* database and code for plotting this images may be found in our repository^6^.

Floods are among the most destructive extreme weather events—between 1995 and 2015, over 2.2 billion people were affected by floods comprising 53% of the total of people affected by all weather-related disasters^1,2^. Situational awareness on the ground is crucial for effective disaster response, and, today, satellite imagery is one of the most important sources of this information^3^. Both passive optical (multi-spectral) and synthetic-aperture radar (SAR) imagery are routinely used to determine flood extent and further derived products^4^ (Fig. 1).

Some regions, like the USA, Europe and Japan have access to high-quality imaging resources from defence organisations and commercial satellite operators through domestic space agencies (i.e., NASA, ESA, JAXA). However, several of the worst flood-affected regions are in developing countries: of the top 20 countries by disaster mortality in proportion to their population for the years 1990–2017, the top five are low or lower-middle-income countries, and only five are upper-middle income^5^.

Many of these countries have almost no means of getting access to higher quality imaging resources via domestic channels. To address this, organisations such as the International Charter “Space and Major Disasters”^7^, initiated by the European Space Agency (ESA), liaise with space agencies and associated commercial organisations to produce free high resolution maps for end-users in the field. Despite best efforts it can take many days to provide actionable information, mainly due to image down-linking and subsequent image analysis^8^. Commercial organisations are able to provide the highest-frequency (daily) and highest-resolution (sub-metre) images, but their satellites must also be tasked and their images may only be freely available for a limited period of time during disasters via the International Charter Space and Major Disasters. ESA’s Copernicus program^9^ provides open data globally at 10 m resolution, but the optical component, Sentinel-2 (S2)^10^, has a revisit time of five days at the equator and two to three days at mid-latitudes. This leads to wait periods much longer than two days in areas such as central Africa where alternatives for rapid data capture can be limited.

In this work we investigate how a constellation of small, inexpensive, nano satellites assembled from commercial off-the-shelf (COTS) hardware, also known as CubeSats^11^, could be used for disaster response, using flooding as a case study. The main advantage of using CubeSats is an improved revisit time through larger constellations of satellites. Commercial organisations like Planet Labs, Inc. (California, USA) have demonstrated the potential for large fleets of low-cost satellites for Earth observation (EO), though their data are only freely available in small quantities. Tens of CubeSats similar to ESA’s FSSCat mission^12^ could be launched for the cost of a single conventional Earth observation satellite, with 30 CubeSats reducing the nominal revisit time from five days to around eight hours for a similar cost. However, CubeSats can have very limited downlink bandwidth, on the order of 1–10 Mbps^13^, compared to around 0.5 Gbps for S2^10^ (Downlink is communication from the satellite back to a ground station on Earth. It is very constrained for CubeSats because the satellite itself must act as a transmitter). In addition to this, there is a cost associated with downlinking data which is proportional to the transfer size, desired frequency and availability of ground stations.

Constrained downlink budgets are a common problem in space science and can be addressed using on-board processing for both targeted data acquisition and filtering. Examples include autonomously identifying science targets on Mars^14,15^ and discarding cloud-obscured imagery on NASA’s EO-1 satellite with the Autonomous Sciencecraft Experiment (ASE)^16,17^. On-board flood detection and mapping (an image segmentation task) has also been proven with ASE^18^ using Hyperion, a 220-band hyperpsectral camera with a 30 m ground sample distance. The output was limited by the computational capability of the satellite and only a small 7.7 $\times$ 30 km region in the centre of the field of view could be processed using 12 of 220 bands. Flood detection was based on simple band thresholds, and an event was triggered based on the number of water pixels in a region compared to a baseline; the combination of three on-board classifiers achieved accuracies of 70–85.6%.

We propose to take this approach further leveraging modern deep learning^19^ algorithms, to perform multiclass segmentation with high accuracy, on-board of very cheap satellite hardware. In order to demonstrate feasibility, we optimise our application for ESA’s $\Phi$Sat-1, part of FSSCat^20^—a technology demonstrator mission— launched at 2nd of September 2020. Among other sensors, FSSCat carries a Cosine HyperScout 2 49-band hyperspectral camera (70  m ground sample distance at 500 km) which integrates an Intel Movidius Myriad2 vision processing unit (VPU) as a co-processor for performing on-board computer vision and neural network inference^12,21^. FSSCat is a 3 $\times$ 2U CubeSat, with HyperScout taking up 1U (10  $\times$  10  $\times$  11 cm) of space. The first machine learning application deployed on the satellite is a cloud detection model^22^ similar to the system used on EO-1.

Using the on-board VPU to perform segmentation, an output two-bit flood map (up to four classes) would reduce the amount of data being down-linked by a factor of 100 (assuming 49 12-bit channels). Since segmented regions tend to be quite large and continuous, there could likely be further savings via simple compression methods like run-length encoding^23^. Our models are trained on a new extensive dataset of human-annotated flood maps covering more than 100 flood events and tested on five independent events from different locations around the globe. We made this dataset available at https://tinyurl.com/worldfloods. While we address flooding in this paper, satellites with on-board capability are attractive as they can potentially be re-targeted for multiple diverse missions, and on-board models can be improved over time if their weights are small enough.

The contributions of this paper are as follows:

1. We introduce a new dataset—*WorldFloods*—that combines, in “machine-learning ready form”, several existing databases of satellite imagery of historical flood events. The dataset contains pairs of Sentinel-2 images and flood extent maps covering 119 global flood events.
2. Using this dataset, we train several convolutional neural network (CNN) architectures for flood segmentation and compare their performance against standard baselines: linear models and a per-image optimal threshold on the normalised difference water index (NDWI)^24^.
3. We show that our models can process large volumes of hyperspectral data, yet fit the constraints of hardware deployed on the satellite. Specifically we report results on the on-board co-processor Intel Movidius Myriad2, which we found was able to process a 12 MP image in less than a minute.

## Background

### Flood mapping

Water mapping, of which flood mapping is a special case, is a semantic segmentation task (also called land cover classification in remote sensing) that has been studied for decades. A simple approach to water mapping is to compute indices like the normalised difference water index (NDWI)^24^ which exploits the difference in absorption of light by water bodies between the green and the near infrared part of the electromagnetic spectrum. However, this method can perform poorly because the spectral profile of flood water varies widely due to the presence of debris, pollutants and suspended sediments^25^. As a result, the main challenge with using indices at a global scale is that the threshold for water retrieval must be tuned per environment. SAR images (e.g., Sentinel-1) are commonly used for water retrieval as they are not affected by cloud cover^26,27^, severe weather and lighting conditions. Since calm water strongly reflects radar wavelengths away from the receiving antenna (specular reflection), image thresholding is a straightforward way to identify water regions by their very low backscatter intensity. However, the presence of waves or wind causes significant backscatter, which can make inland water harder to identify. In addition, flooding in urban areas^28^ is difficult to map due to multiple reflections by buildings and taller vegetation which produces an increase in backscatter. Additionally, as SAR is an active sensing technique with a high power requirement (e.g. Capella constellation, 600 Watts for transmission^29^), deployment on a small satellite is challenging; we therefore limit the scope of this paper to passive optical sensors, but we do use some training data derived from Sentinel 1 imagery.

More sophisticated segmentation techniques include rule-based classifiers^18,25^ which use a fixed or tuned threshold on indices or individual bands; classical supervised machine learning^3^; and recently deep learning^30–33^. Among deep learning methods, fully convolutional neural networks (FCNNs)^34^ produce state-of-the-art results in image segmentation tasks with fast inference time; they are thus the model proposed for this application.

### Hyperspectral image processing

One of the inherent difficulties of targeting a satellite that has yet to be launched is that no real-world orbital data are available. This problem is usually addressed by using data from a similar satellite and accounting for known differences in spectral sensitivity^35^. However, in the case of $\Phi$Sat-1, the problem is exacerbated as there are very few satellites with hyperspectral sensors and archival data are similarly limited^36,37^. Notably HyperScout-1 has been flown in space, on the GOMX-4B mission, but data from this mission are not publicly available^38^. Other aerial missions like AVIRIS (a NASA-modified U2 aircraft)^36,39^ have a larger public archive, but these images are mostly limited geographically to the USA. Since we need labelled data, we have the additional constraint that we rely on serendipitous image acquisition coinciding with flood events.

The images that HyperScout-2 produces are relatively large—45 visible channels and four thermal infrared channels with a dynamic range of 12-bits per pixel. The output image has a spectral resolution of 15 nm over a range of 400–1000 nm. HyperScout-2 is a push-broom sensor; a nominal 2D frame represents approximately a 200 km by 300 km swath at a nominal orbital height of 500 km^38^. The ground sample distance (GSD) at this altitude is 70  m.

**Figure 2:**
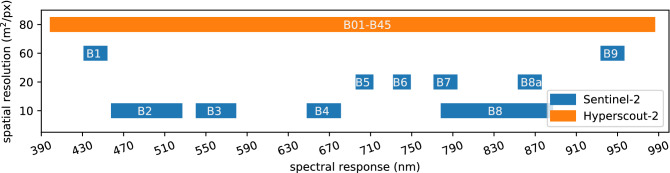
Spatial resolution and spectral response of Sentinel-2 and HyperScout-2 sensors.

We propose to use Sentinel-2 data for model training, which is sensitive to a similar wavelength range, but with fewer bands. S2 spatial resolution varies for each spectral band from 10 to 60  m. In order to produce a model for HyperScout-2 images we follow an approach similar to two recent studies^40,41^ which demonstrate models that show some generalisation to multiple sensors. In particular, we select the bands of Sentinel-2 that are common to HyperScout-2 (shown in Fig. 2) and reduce the spatial resolution of Sentinel-2 images to 80  m using bilinear interpolation. In addition, HyperScout-2 and $\Phi$Sat-1 are expected to have a worse signal-to-noise ratio compared to Sentinel-2 due to its reduced size and poorer direct georeference. In order to account for this, our models are trained with degradatations in form of Gaussian noise, channel jitter (translational offsets) and motion blur. These degradations are implemented as data augmentation functions^42,43^.

## Methods

### Flood segmentation

Given a satellite image (with or without a flood), we wish to label each pixel as water/flood or land. As always with data coming from an optical sensor, we also have to deal with the problem of obstruction by clouds. Since we are targeting on-board processing, we choose to tackle this by adding a cloud class to the output of the model, so that we can maintain the workflow of a single pass over the image. Our models therefore have three output classes (land, water/flood and cloud), requiring two bits of data per pixel to store. Note our model does not distinguish water and flooded pixels; however we report segmentation results on flood and permanent water pixels using the JRC yearly permanent water layer^44^.

### *WorldFloods* dataset

The development and evaluation of flooding response systems has been constrained so far by use of trusted, authoritative or validated datasets that are also often of limited geographical scope, with most studies only considering a single or very few flood events^33,45^. It is unclear whether such models would accurately generalise to the rest of the world due to variations in topography and land cover. To address this we collated a new global dataset called *WorldFloods*, which we believe is the largest collection of its kind.

**Figure 3:**
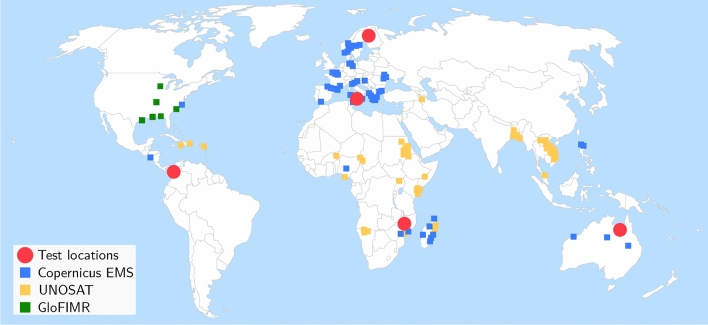
Locations of flood events contained in *WorldFloods*. Blue, orange and green areas denote Copernicus EMS, UNOSAT and GloFIMR data, respectively. Red circles denote test regions. Basemap credit: http://www.simplemaps.com.

*WorldFloods* contains 422 flood extent maps created by photo-interpretation either manually or semi-automatically, where a human validated machine-generated maps. A flood extent map is a vector layer (shapefile) derived from a satellite image with polygons indicating which part of that image has water (in some cases it distinguishes between flood water and permanent water and in other cases it does not); we assigned a date to each flood extent map which corresponds with the date of acquisition of the original satellite image that was used to derive it. Each flood extent map belongs to a flood event hence a flood event could have several flood maps which may cover different areas of interest or different days of the same area in the same flood event; in total the dataset covers 119 floods events that occurred between November 2015 and March 2019. We sourced all maps from three organisations: the Copernicus Emergency Management Service (Copernicus EMS)^46^, the flood portal of UNOSAT^47^, and the Global Flood Inundation Map Repository (GLOFIMR)^48^. The geographical distribution of flood maps is shown in Fig. 3.

**Figure 4:**
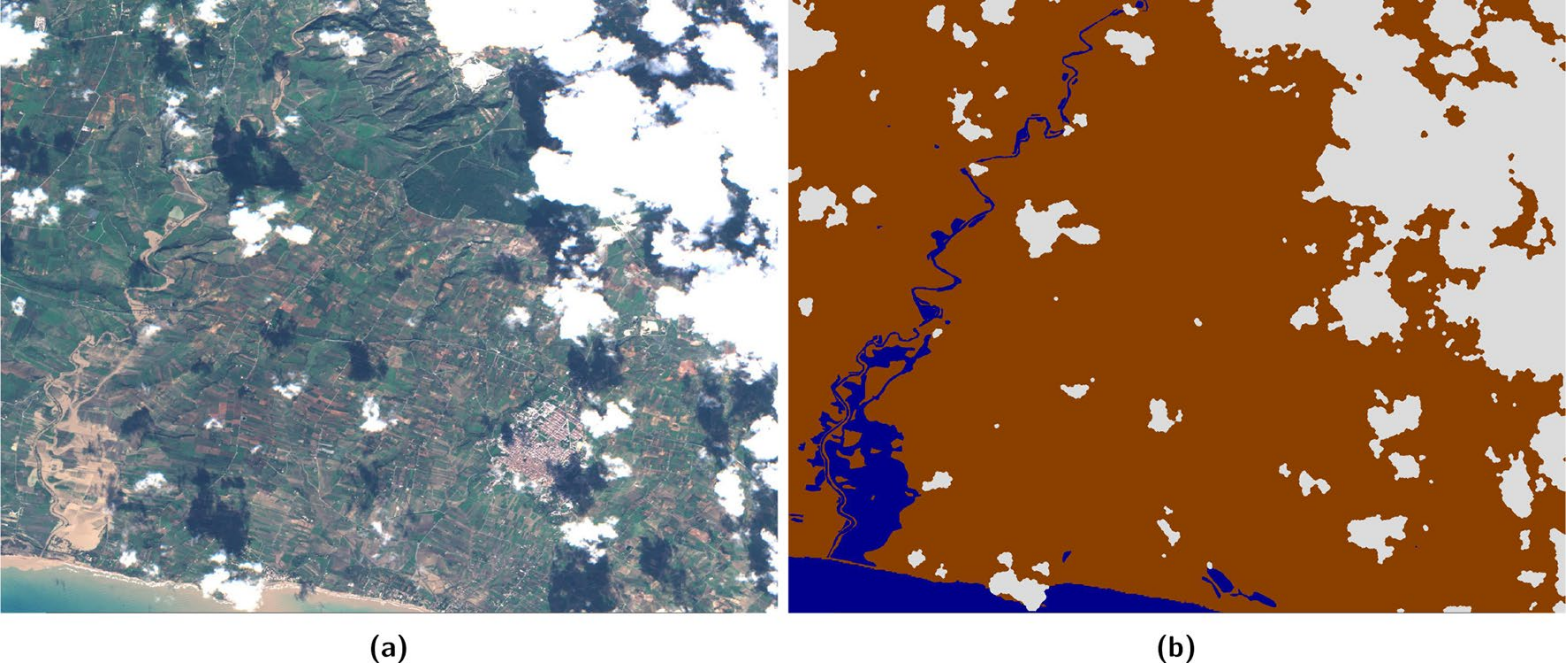
(**a**) Sentinel 2 RGB bands and (**b**) associated labelled map (land/brown, water/blue, cloud/white) over Porto Palo (Sicily) derived from Copernicus EMS 333 activation. Cloud mask obtained automatically with s2cloudless^49^. Base image and reference labels are included in the *WorldFloods* database and code for plotting this images may be found in our repository^6^.

For each flood event we provide the raw 13-band S2 image closest in time after the event, and rasterised *reference labels* (cloud, water and land) at 10  m resolution. (We explicitly avoid the term *ground truth* as labels are derived manually or semi-automatically by photo-interpretation and have not been validated by ground measurements). S2 images were downloaded from the Google Earth Engine^50^; S2 bands with spatial resolution larger than 10  m were resampled to 10  m using nearest neighbours interpolation. We generated cloud masks using s2cloudless^49^. The dataset contains in total more than 12 Gigapixels of labeled data which occupies around 266 GB of disk space. Figure 4 shows an example of S2 image and derived reference labels for a flood that occurred in Central-West Sicily in November 2018.

**Figure 5:**
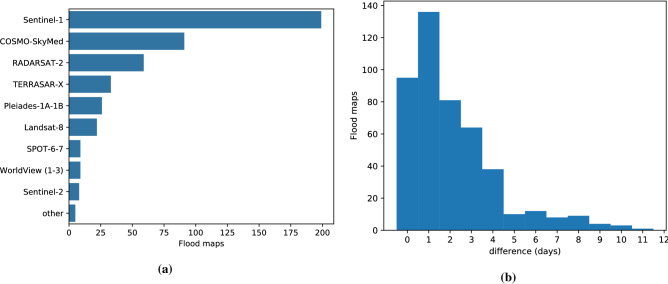
(**a**) Satellite used to derive each flood map in the *WorldFloods* data set. ‘Other’ satellites (all optical): GeoEye-1, PlanetScope, Earth Observing (EO)-1. (**b**) Difference in days between the flood map and the Sentinel-2 image (Sentinel-2 image is always posterior in time to the flood map).

We manually validated the data to account for gross errors such as missing water bodies or invalid intensities. In some cases, missing water bodies were filled using the permanent water bodies dataset^44^ available from the Google Earth Engine^50^ (we also use this data to differentiate flood and permanent water in the results). Nevertheless, there are still mislabeled pixels specially in narrow streams, partially inundated crop fields and in the borders of clouds and water bodies. Some of these errors are caused by temporal misalignment, e.g., the closest S2 image may have been acquired some days after the map was produced. This happens, as is frequently the case, if the flood extent map was generated based on a satellite image other than S2. Figure 5 shows, on the left, the satellites used to derive each flood map and on the right, the difference in days between the flood extent map and the next S2 overpass. As we can see, most of the flood extent maps where generated from radar imagery and most images are acquired within five days which suggests that the earliest available re-visit is used if available.

While including flood extent maps and S2 images from different days introduces label noise, this allows us to use a much larger training set than if we were restricted to images where the flood map was generated from S2. We were motivated by results from the authors of SEN12MS^51^ who trained global high resolution (10 m) segmentation models using cloud-free S2 imagery and low resolution labels derived from MODIS (500 m), achieving 63–65% overall accuracy despite the coarseness of the available ground truth labels. In our results section we experimentally validate that this trade-off is justified for our dataset; that is, we achieve better segmentation results on a clean test set when we include these noisy labels in our training than if we restrict the training set to clean images.

Models trained on noisy labels in the training may appear to perform well, but it is important to ensure that the test set provides a clean measurement of the true performance of our system. In this direction, we manually selected test images from flood extent maps that were derived from S2 images which had no temporal misalignment. In addition, we visually inspected those images and fixed minor errors to improve the quality of their segmentation masks. To avoid data leakage, there was no spatial overlap between flood maps in the test set and the training and validation sets. Additionally, other flood extent maps from same flood events in the test set have also been removed from the training and validation sets. Table 1 shows the training, validation and test set statistics; there is a strong class imbalance in the training dataset with less than 3% of pixels belonging to the water class. From those, less than 50% are classified as permanent water in the JRC permanent water product^44^. The low occurrence of water pixels in the train dataset is because there is a high presence of clouds in the training data. Cloud occurrence in the validation and test sets is lower to provide more meaningful results of flood segmentation.

**Table 1:** General statistics of the training, validation and test splits of the *WorldFloods*  dataset. Since raw images from S2 can be many megapixels in size, we tile each image into 256-pixel square patches. The training set distribution has a higher percentage of cloudy pixels compared with the validation and test datasets; this is because we were interested in distinguishing water/flood pixels whereas detecting clouds is a byproduct of the model.

Dataset	Flood events	Flood maps	256x256 patches	Water pixels (%)		Land pixels	Cloud pixels	Invalid pixels
				Flood	Permanent$^{\dag }$	(%)	(%)	(%)
Training	108	407	182,413	1.45	1.25	43.24	50.25	3.81
Validation	6	6	1132	3.14	5.19	76.72	13.27	1.68
Test	5	11	2029	20.23	1.16	59.05	16.21	3.34

$^{\dag }$ Permanent water obtained from the yearly water classification product of Pekel et al.^44^ available at the Google Earth Engine^52^.

## Results

In order to demonstrate that a FCNN-based flood detection model can segment floods accurately and could be deployed on $\Phi$Sat-1, we first train FCNN models on *WorldFloods* at its original resolution (10  m). We then train models on *degraded* imagery, mimicking the resolution of HyperScout-2 (80  m) by resampling the S2 images using bilinear interpolation and also by using only the overlapping bands between the sensors. Afterwards, models trained over the entire *WorldFloods* dataset are compared with models trained using only flood maps derived from Sentinel-2. Finally, we verify our trained (degraded) models can be run on a Intel Movidius Myriad2 chip and measure the processing speed; we use an Intel Neural Compute Stick v1 connected to a Raspberry Pi 3B+. Models tested on the Intel Movidius Myriad2 chip use all available S2 bands, in comparison to the cloud detection model^22^ which uses three bands selected using Principle Component Analysis (PCA).

We focus on the segmentation accuracy of the water/flood class by measuring precision, recall and the intersection over union (IoU). Since missing flooded areas (false negatives) is more problematic than over-predicting floods (false positives), high recall is preferred to high precision. In practice the IoU is a good compromise if recall is sufficiently high (over 94%); with a lower recall we found that, even with a high IoU, the model misses entire water bodies in several scenes.

As baselines, we use NDWI (S2 band 3 and 8^24^) and a per-pixel linear model (all S2 bands) trained on *WorldFloods*. A range of NDWI thresholds have been suggested in the literature for flood water extraction^24,25,53^we chose 0 for our experiments since it is the most common one. In order to set a stronger baseline, we also report results for the threshold that maximizes the IoU in the test data providing a recall above 94% (threshold − 0.22). This represents the best case performance for the NDWI model. In addition, in order to strengthen the baseline results, the NDWI model assumes perfect cloud masking by using directly the s2cloudless cloud masking model. We compare our baselines to two FCNNs: a simple CNN (SCNN) comprising four convolutional layers (0.26M parameters) and a U-Net (7.8 M parameters)^54^. Although single-pixel classification methods like NDWI are common, we expect that models which can use larger contextual information, such as the extended shape of water bodies, will perform better. Therefore we calculated the receptive field of our models to ensure that larger features are considered during classification. Our smallest model has a receptive field of 9 $\times$  9 pixels (700  $\times$  700 m) which we judged to be sufficient. Details of our SCNN and UNet architectures can be found in the supplementary material for this paper; additionally our implementation and training code is provided in our GitLab repository^6^.

**Figure 6:**
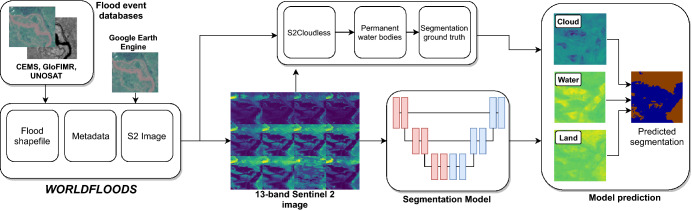
Overview of the model training pipeline used in this work. Note that *WorldFloods* provides images from S2, but reference flood extent maps may have been labelled from other sources, such as radar satellites.

Models were trained from scratch for 40 epochs using all 13 S2 bands with input patches of size 256 $\times$ 256 for 10  m data or 64 $\times$ 64 for 80  m data (2.5 km  $\times$  2.5 km). For data at 80 m resolution we also trained our models using only the 10 overlapping bands between HyperScout-2 and S2 (see Fig. 2). In order to achieve models with high recall we used a cross-entropy loss function that weights each class by the inverse of the observed frequency in Table 1, combined with a Dice loss^55^. Augmentation was applied during training including flips and rotations, per-channel jitter, Poisson (shot) noise and brightness/contrast adjustments. A flowchart showing the training and dataloading process is shown in Fig. 6. Models were tested on full S2 images as described in^56^.

**Table 2:** IoU and recall results for models trained on *WorldFloods*.

	Model	IoU total water	Recall total water	Recall flood water	Recall permanent water
10 m	NDWI (thres -0.22)	65.12	**95.75**	95.53	**99.70**
	NDWI (thres 0)	39.99	44.84	42.43	86.65
	Linear	64.87	95.55	**95.82**	90.75
	SCNN	71.12	94.09	93.98	95.93
	U-Net	**72.42**	95.42	95.40	95.83
80 m	NDWI (thres -0.22)	64.10	94.76	94.57	98.15
	NDWI (thres 0)	39.07	44.01	41.69	84.55
	Linear	60.90	95.00	94.79	**98.58**
	SCNN	68.87	**96.03**	**96.11**	94.76
	U-Net	**70.22**	94.78	94.85	93.50
80 m HyperScout-2 overlapping bands	NDWI (thres -0.22)	64.10	**94.76**	94.57	**98.15**
	NDWI (thres 0)	39.07	44.01	41.69	84.55
	Linear	50.27	80.47	79.69	94.03
	SCNN	**65.82**	94.62	**95.17**	84.99
	U-Net	65.43	94.59	**95.17**	84.44

Bold values indicate highest metric value for each resolution and band combination.

**Figure 7:**
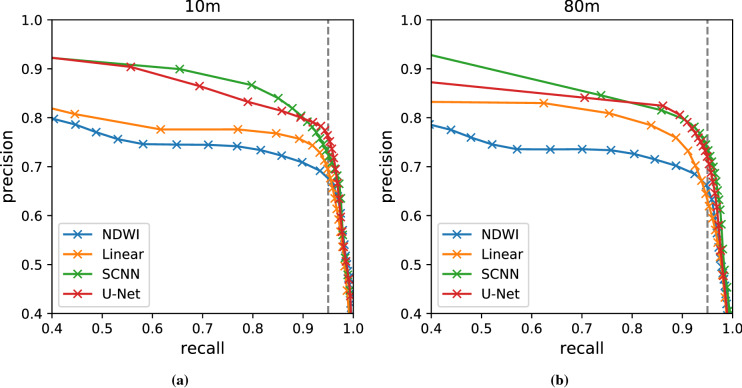
Precision–recall curves of different models trained on (**a**), the Sentinel-2 original resolution (10 m) and (**b**), in the degraded resolution of HyperScout-2 (80 m). In gray 95% recall threshold.

Table 2 shows the metrics for the different models and baselines. Specifically, we show IoU and recall for the water class (total water) as well as the recall stratified for flood and permanent water. Permanent water classification comes from the JRC permanent water layer^52^. Our three models (Linear, SCNN and UNet) all have a recall above 94%; NDWI with the threshold at zero generalises poorly, we suspect due to water with suspended matter. FCNN models performed best although there was only a small increase in performance between SCNN and U-Net, despite U-Net having 30 $\times$  more parameters. The drop in performance from 10 to 80  m is around two points for FCNN models which is acceptable taking into account that the spatial resolution is eight times worse. There is also a significant drop in performance when only the 10 overlapping bands of HyperScout-2 and S2 are used (bands B1 to B9) suggesting that the short-wave infrared (SWIR) bands of S2 (B10–B12) have high predictive power for water. This is expected since water reflectance is very low in the SWIR whereas soil and vegetation reflectance is significantly higher^57^. Figure 7 shows the precision and recall for different thresholds on the total water class; again, our trained models beat NDWI and larger models tend to perform better.

**Figure 8:**
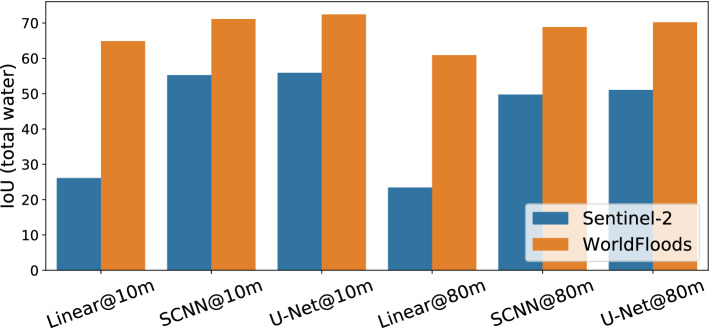
Performance of models trained with all *WorldFloods* flood maps compared with models trained only with flood maps derived from Sentinel-2.

Figure 8 shows the results of the models trained on the *WorldFloods* training dataset against models trained on clean S2–labelled data alone (Fig. 8). Results for the clean S2 labeled data have been computed by cross validation leaving one flood event out from the *WorldFloods* test dataset (details on this procedure and results for each flood event can be found in the supplementary material). We found that training using all data was better than training on S2-labelled data alone. Our hypothesis is that although reference labels from non-S2 satellites may be noisier, when considering the dataset in aggregate, this noise becomes less significant as most pixels are labelled correctly. This result also lends support to our argument that temporal misalignment between labels and imagery in our dataset was not significant. Similarly, this robustness should also extend to noisy ground truth which is semi-automatically labelled by humans.

**Figure 9:**
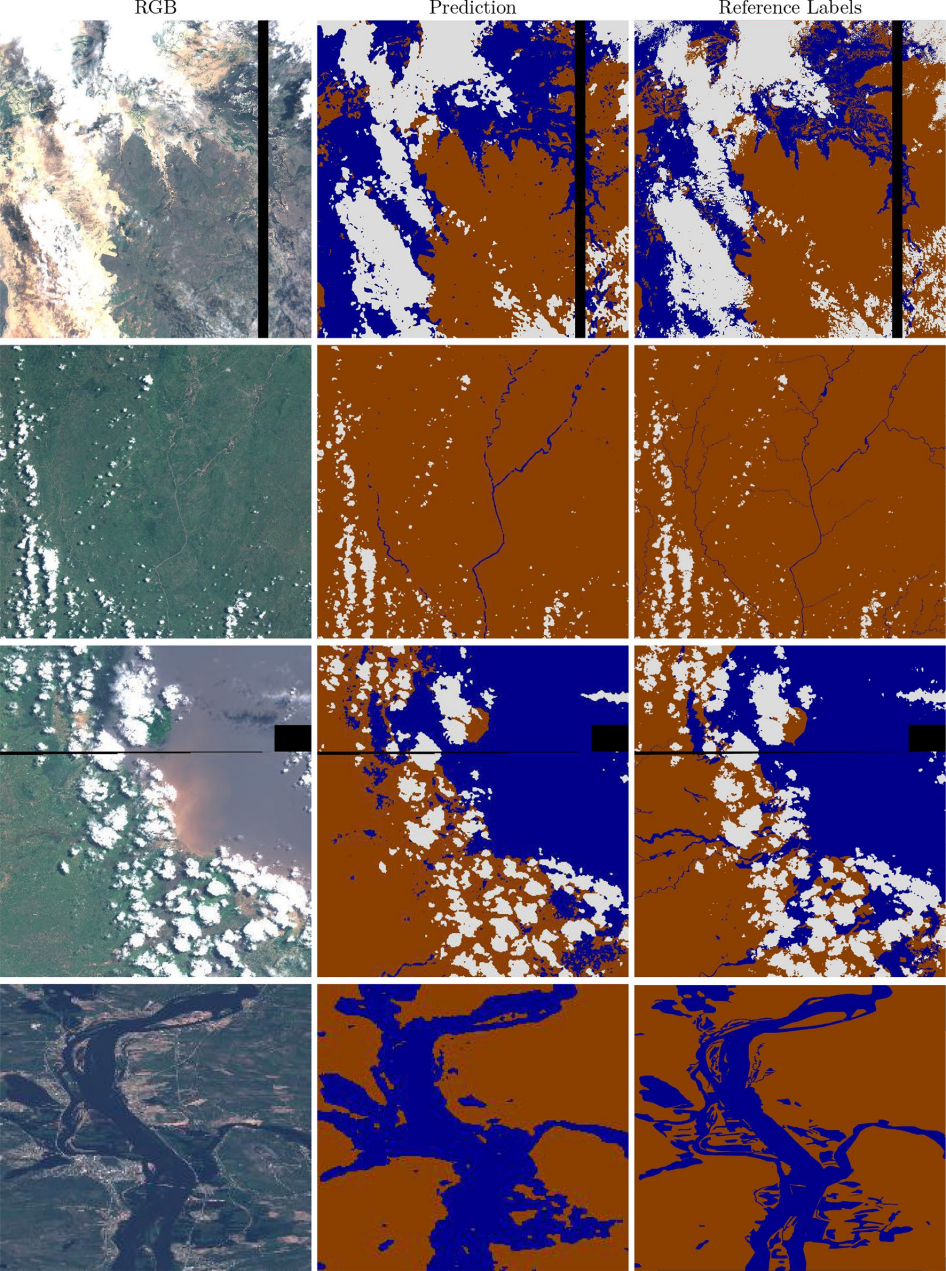
Segmentation results of degraded models (SCNN 80  m) run on Myriad 2 device. Sentinel 2 imagery and Copernicus EMS mapping products are provided as public domain. Base images and reference labels are included in the *WorldFloods* database and code for plotting these images may be found in our repository^6^. Colours are as follows: brown/land, blue/water, white/cloud.

The SCNN model was selected for testing on the Myriad 2 chip due to its similar accuracy, but lower computational footprint, compared to UNet (1 FLOPS vs 2.68 FLOPS for a 64 $\times$  64 $\times$  13 input). Figure 9 shows some example images segmented using the Myriad2. This model segments a 12 MP image—approximately the size acquired by HyperScout-2—in less than one minute, accounting for data transfer between the computer and the accelerator development board via a USB connection. We assume that the power required to downlink data is comparable to that of data processing (2.5 W for the Myriad2). Using a radio with a bandwidth of 10 Mbps, a 1GB image would take 13 minutes to transfer. Therefore we can reduce image transmission power consumption by an order of magnitude at least. On a fully integrated platform like a satellite, we would expect lower latency for data transfer and hence a slightly faster overall processing time.

In general, our models tend to over-predict water content; a common failure mode is to identify dark regions as water. False positives are mostly clustered in the surroundings of water bodies and in cloud shadows (see Fig. 9). For further work we are exploring other methods to improve this, for example by adding another input channel with elevation.

## Discussion and conclusions

The current proliferation of open-access satellite data complemented by imagery from commercial satellite operators has still only limited impact on assisting disaster response, primarily because of relatively low revisit times and long delays between image acquisition and product delivery. Here we propose a technical concept study for in-orbit flood mapping using low-cost hardware with machine learning capability to reduce the amount of data required to be downlinked. This concept will enable the use of large cubesat constellation to reliable monitor environmental phenomena such as flooding with high temporal resolution.

We have demonstrated that accurate flood segmentation in orbit is feasible to perform using low resolution images and available hardware. Our models outperform standard baselines and are favourably comparable to human annotation, while being efficiently computable with machine learning hardware on-board the current $\Phi$Sat-1 technology demonstrator as well as future missions.

Recent works^58,59^ have shown good performance of spectral indices such as NDWI for water detection on specific survey areas. In our experiments we see that our “best case” tuned NDWI results are also a strong baseline. However there are still examples where a fixed threshold in an image will incorrectly retrieve buildings and cloud shadows as water. Therefore we expect NDWI to perform well in some cases (in our dataset, Finland, for example) and poorly in others, which is perhaps reflected in our aggregated results (see table 3 in supplementary materials for the results for each flood event). Compared to previous works on flood detection^33,45^, we have reported results on a wide range of geographical areas paying special attention to data leakage^60^. For our application global generalisation is critical since its intended use is to automatically provide segmentation masks instead of heavier hyper-spectral images.

Downlinking only segmentation masks instead of complete images is not exempt from drawbacks. Firstly, the quality of the downloaded data only depends on the accuracy of the model. In other words, an erroneous segmentation can not be fixed on the ground since the original hyperspectral information is lost. This could be alleviated by periodically downlinking full images to assess and improve the segmentation algorithm’s quality. The newly gained data could be added to the training dataset or even apply domain adaptation^61^ to boost the segmentation networks. Secondly, by discarding the image, we lose information that could be used for advanced analysis. Hyperspectral information could be used to assess the presence of pollutants in the flood water. In this case, the segmentation masks could be used to guide the retrieval of relevant pixels. Guiding the retrieval of cloud free images is the current operational application onboard the $\Phi$Sat-1 satellite^22^.

One of the contributions of this work is the release of the *WorldFloods* database alongside this paper, which we hope will serve as a useful tool to foster further research in disaster response. We are pleased to write that this approach is being increasingly explored - while this work was being prepared for publication, several other ‘machine learning’ ready datasets for segmentation from satellite imagery have been published; Rambour et. al.^62^ demonstrated flood detection on time series of SAR and optical data, making their dataset publicly available, Bonafilia et. al.^63^, who focus on Sentinel 1 data, but provide more detailed labels that we had available to us here and Nemni et al.^64^ who has also made their dataset publicly accesible. The approach we explore here, of producing a ’machine learning ready’ dataset as well as a concrete algorithm, has also been recently explored for other areas of disaster response^65^, and we hope to see this continue.

## Supplementary information

Supplementary Information

## Data Availability

We are releasing the *WorldFloods* database alongside this paper at https://tinyurl.com/worldfloods. Users of this dataset should be aware of the varying quality of the reference labels that is pointed out in the paper; specifically some labels in the training and validation datasets have significant errors. In general the quality of the test dataset labels are higher and test images were curated to facilitate more accurate model evaluation. We hope to address any remaining label quality issues in future work. We provide a GitLab repository with our model architectures, model checkpoints and training/benchmarking code at: https://gitlab.com/frontierdevelopmentlab/disaster-prevention/cubesatfloods.

## Duplicate publication statement

A short summary of some material in this paper was previously presented at the Humanitarian Aid and Disaster Response (HADR) workshop at NeurIPS 2019 (Vancouver)^66^. The pre-print was peer-reviewed for inclusion in the workshop, which is not archival and does not form part of the NeurIPS conference proceedings. This paper provides substantially more information and addresses several aspects of the dataset that were not discussed in the workshop paper (such as the the effects of training on S2 only, the effect of temporal misalignment and stratification of results on flood and permanent water). We have also extensively curated the training and testing sets and we include a more thorough discussion of the context of our work, flood mapping, and the satellite we have targeted for deployment. Alongside this paper, we also release the *WorldFloods* dataset as well as the code used to train and benchmark our models.
