# Supplementary material for: Towards global flood mapping onboard low cost satellites with machine learning
Source: Sci Rep. 2021 Mar 31;11:7249. doi: 10.1038/s41598-021-86650-z (PMC8012608; doi:10.1038/s41598-021-86650-z)
Supplement: Supplementary file 1 — Supplementary Information [file 41598_2021_86650_MOESM1_ESM.pdf]

Towards Global Flood Mapping Onboard Low Cost Satellites with Machine Learning

Gonzalo Mateo-Garcia[1,+*], Joshua Veitch-Michaelis[2,+], Lewis Smith[3,+], Silviu Oprea[4], Guy Schumann[5,6], Yarin Gal[3], Atılım Güneş Baydin[3], and Dietmar Backes[7]

[1]Universidad de Valencia
[2]Liverpool John Moores University
[3]University of Oxford
[4]University of Edinburgh
[5]University of Bristol
[6]RSS-Hydro, RED
[7]University of Luxembourg
[*]Correspondence to: Gonzalo.Mateo-Garcia@uv.es
[+]These authors contributed equally to this work

ABSTRACT

This supplementary material briefly describes the neural network architectures used in this work. It also reports the recall and IoU metrics for each of the flood events in the test dataset.

Neural Network architectures

SimpleCNN

SimpleCNN is a simple CNN with four convolutional layers, each followed by a Rectified Linear Unit (ReLU) activation. The output is a 3 channel image the same shape as the input. Softmax is applied at the final layer to convert the network output into classification proabilities. Table 1 lists the details of each layer and a schematic diagram of the architecture is shown in Figure 1.

Name	Operation	Depth	Kernel	Stride	Pad
C1	Conv2D	64	3	1	1
	ReLu	-	-	-	-
	Conv2D	64	3	1	1
	ReLu	-	-	-	-
D1	Conv2D	128	3	1	1
	ReLu	-	-	-	-
	Conv2D	128	3	1	1
	ReLu	-	-	-	-
	Conv2D	3	1	1	1
Out	Softmax	-	-	-	

Table 1. SimpleCNN Layer Architecture

UNet

UNet is one of the most commonly used segmentation architectures. It comprises of two stages: an encoder and decoder. The encoder performs convolutions followed by maxpool operations to progressively downsample the input. Conversely, the decoder performs convolutions followed by 2x upsampling using bilinear interpolation. The network is symmetric so that the output size is the same as the original image. Skip connections are used to provide local information during the upsampling step. Softmax is applied at the final layer to convert the network output into classification proabilities.

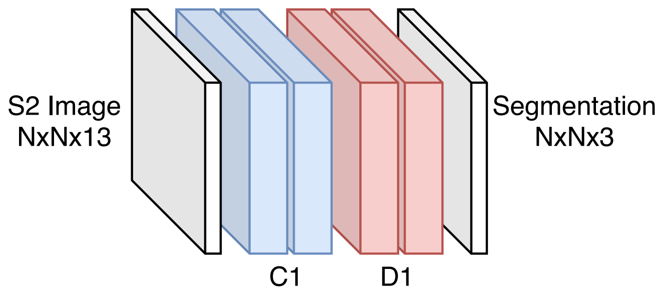

Figure 1. SimpleCNN model diagram. Layer groups are listed in more detail in Table 1. Nominally for the *WorldFloods* dataset, $N = 256$px or 64px when used for simulated on-board processing.

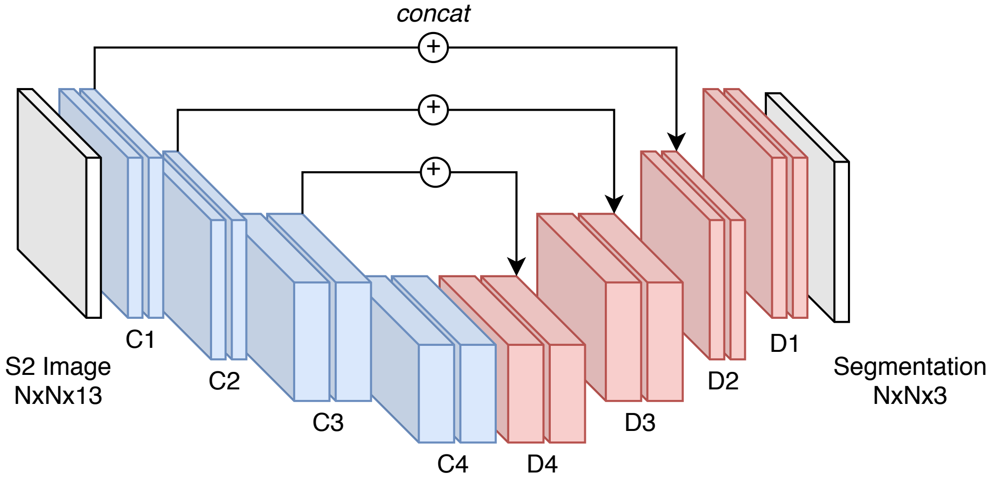

Figure 2. UNet model architecture. Encoding layers are in blue, decoding layers are in red. UNet is characterised by skip connections between the encoder and decoders. Layer groups are listed in more detail in Table 2. Nominally for the *WorldFloods* dataset, $N = 256$px or 64px when used for simulated on-board processing.

Metrics for test floods events

Table 3 shows the general statistics of the flood maps in the test dataset. Table 4 shows the IoU and recall metrics of the water class for each flood event in the test dataset. We report results for the different models at 10 m and 80 m resolutions. The *training dataset* column indicates which dataset was used for training the models. In that column, 'WorldFloods' corresponds to the *WorldFloods* training dataset whereas 'Sentinel-2' corresponds to models trained *by-leave-one-flood-out* in the *WorldFloods* test dataset. This procedure corresponds to: for a given flood event in the *WorldFloods* test dataset the flood extent maps are partitioned on two subsets. The *test subset* is formed by flood maps from that flood event and the *train subset* consists of flood maps NOT from that flood event. Models are trained on the *train subset* and tested on the *test subset* for each flood event in the *WorldFloods* test dataset. Metrics shown in table 4 corresponds to those test results.

Name	Operation	Depth	Kernel	Stride	Pad
C1	Conv2D	64	3	1	1
	ReLu	-	-	-	-
	Conv2D	64	3	1	1
	ReLu	-	-	-	-
	Maxpool	-	2	2	0
C2	Conv2D	128	3	1	1
	ReLu	-	-	-	-
	Conv2D	128	3	1	1
	ReLu	-	-	-	-
	Maxpool	-	2	2	0
C3	Conv2D	256	3	1	1
	ReLu	-	-	-	-
	Conv2D	256	3	1	1
	ReLu	-	-	-	-
	Maxpool	-	2	2	0
C4	Conv2D	512	3	1	1
	ReLu	-	-	-	-
	Conv2D	512	3	1	1
	ReLu	-	-	-	-
	Upsample	-	-	-	-
	Concat C3	-	-	-	-
D3	Conv2D	256	3	1	1
	ReLu	-	-	-	-
	Conv2D	256	3	1	1
	ReLu	-	-	-	-
	Upsample	-	-	-	-
	Concat C2	-	-	-	-
D2	Conv2D	128	3	1	1
	ReLu	-	-	-	-
	Conv2D	128	3	1	1
	ReLu	-	-	-	-
	Upsample	-	-	-	-
	Concat C1	-	-	-	-
D1	Conv2D	64	3	1	1
	ReLu	-	-	-	-
	Conv2D	64	3	1	1
	ReLu	-	-	-	-
	Conv2D	3	1	1	1
Out	Softmax	-	-	-	

Table 2. Unet Layer Architecture

Flood event	Flood maps	256x256 patches	Water pixels (%)		Land pixels (%)	Cloud pixels (%)	Invalid pixels (%)
			Flood	Permanent†			
EMSR286 (Colombia)	2	83	3.34	0.16	43.95	50.17	2.37
EMSR333 (Italy)	3	45	2.57	1.75	75.29	14.38	6.01
EMSR286 (Australia)	2	946	36.22	1.34	39.30	19.16	3.98
EMSR347 (Malawi)	3	919	6.76	0.59	79.15	10.73	2.78
EMSR284 (Finland)	1	36	14.39	11.41	74.14	0.00	0.06

† Permanent water obtained from the yearly water classification product of Pekel et al.[?] available at the Google Earth Engine[?].

Table 3. General statistics of the flood maps in the test dataset.

Flood event	Resolution	Model	Training dataset	IoU Total Water	Recall Total Water	Recall Flood Water	Recall Permanent Water†
EMSR284 (Finland)	10m	NDWI (thres -0.22)	-	76.82	94.71	91.05	99.32
		NDWI (thres 0)	-	**82.06**	87.34	78.52	98.46
		Linear	Sentinel-2	28.73	99.64	99.37	99.99
			WorldFloods	81.31	94.36	90.41	99.36
		SCNN	Sentinel-2	60.95	98.53	97.51	99.82
			WorldFloods	74.66	97.01	94.72	99.89
		U-Net	Sentinel-2	62.27	96.18	94.04	98.88
			WorldFloods	77.72	96.35	93.59	99.84
	80m	NDWI (thres -0.22)	-	71.90	90.68	84.26	98.83
		NDWI (thres 0)	-	**74.96**	82.63	70.53	98.01
		Linear	Sentinel-2	25.82	99.96	99.92	100.00
			WorldFloods	75.83	91.41	85.56	98.85
		SCNN	Sentinel-2	69.58	97.88	96.42	99.74
			WorldFloods	67.88	99.05	98.31	100.00
		U-Net	Sentinel-2	68.01	98.43	97.32	99.84
			WorldFloods	65.14	99.39	98.90	100.00
EMSR286 (Australia)	10m	NDWI (thres -0.22)	-	65.01	98.13	98.07	99.83
		NDWI (thres 0)	-	36.47	41.73	40.26	81.40
		Linear	Sentinel-2	49.07	98.70	98.82	95.51
			WorldFloods	64.22	98.20	98.76	83.02
		SCNN	Sentinel-2	53.69	99.10	99.19	96.55
			WorldFloods	70.28	96.51	96.66	92.53
		U-Net	Sentinel-2	54.17	98.73	99.36	81.88
			WorldFloods	**71.78**	98.16	98.37	92.38
	80m	NDWI (thres -0.22)	-	64.50	97.67	97.65	98.16
		NDWI (thres 0)	-	35.67	41.00	39.63	77.87
		Linear	Sentinel-2	40.53	99.42	99.49	97.47
			WorldFloods	61.31	97.88	97.86	98.61
		SCNN	Sentinel-2	49.00	99.22	99.19	99.99
			WorldFloods	68.40	98.13	98.42	90.29
		U-Net	Sentinel-2	51.24	99.05	99.02	99.98
			WorldFloods	**70.22**	97.55	97.90	88.30
EMSR286 (Colombia)	10m	NDWI (thres -0.22)	-	56.18	85.53	84.85	99.34
		NDWI (thres 0)	-	71.47	73.02	72.49	83.77
		Linear	Sentinel-2	12.31	95.42	95.20	99.88
			WorldFloods	58.20	84.19	83.47	98.69
		SCNN	Sentinel-2	64.38	91.56	91.23	98.39
			WorldFloods	**83.31**	92.53	92.18	99.59
		U-Net	Sentinel-2	47.82	94.50	94.29	98.74
			WorldFloods	81.47	92.43	92.15	98.27
	80m	NDWI (thres -0.22)	-	49.38	79.96	79.36	92.41
		NDWI (thres 0)	-	64.79	68.46	68.09	75.95
		Linear	Sentinel-2	5.62	100.00	100.00	100.00
			WorldFloods	41.75	78.61	77.81	94.94
		SCNN	Sentinel-2	21.56	96.11	95.95	99.37
			WorldFloods	**65.74**	97.11	96.97	100.00
		U-Net	Sentinel-2	21.97	95.22	95.02	99.37
			WorldFloods	65.36	96.37	96.22	99.37
EMSR333 (Italy)	10m	NDWI (thres -0.22)	-	19.09	81.72	69.48	99.77
		NDWI (thres 0)	-	31.14	41.13	6.08	92.80
		Linear	Sentinel-2	6.30	99.65	99.55	99.81
			WorldFloods	25.13	87.28	79.14	99.28
		SCNN	Sentinel-2	15.21	98.59	97.89	99.62
			WorldFloods	**51.68**	87.31	78.97	99.60
		U-Net	Sentinel-2	16.60	98.67	97.94	99.75
			WorldFloods	50.37	82.77	71.36	99.59
	80m	NDWI (thres -0.22)	-	18.20	77.33	62.82	99.68
		NDWI (thres 0)	-	30.68	40.42	5.09	94.81
		Linear	Sentinel-2	5.07	100.00	100.00	100.00
			WorldFloods	22.42	77.96	64.05	99.37
		SCNN	Sentinel-2	17.28	96.17	94.02	99.47
			WorldFloods	**40.64**	87.04	78.83	99.68
		U-Net	Sentinel-2	15.44	97.29	95.81	99.58
			WorldFloods	36.01	77.92	63.71	99.79
EMSR347 (Malawi)	10m	NDWI (thres -0.22)	-	69.89	84.41	83.09	99.73
		NDWI (thres 0)	-	52.80	53.25	50.27	87.73
		Linear	Sentinel-2	7.97	99.98	99.98	99.99
			WorldFloods	71.09	82.65	81.17	99.74
		SCNN	Sentinel-2	74.20	85.05	83.76	99.93
			WorldFloods	**76.53**	81.25	79.65	99.74
		U-Net	Sentinel-2	76.95	85.30	84.04	99.89
			WorldFloods	76.41	81.52	79.94	99.74
	80m	NDWI (thres -0.22)	-	66.36	81.44	80.07	97.46
		NDWI (thres 0)	-	52.24	52.71	49.76	87.07
		Linear	Sentinel-2	7.95	99.97	99.97	100.00
			WorldFloods	60.81	81.79	80.37	98.26
		SCNN	Sentinel-2	60.72	85.01	83.90	97.93
			WorldFloods	**74.06**	84.84	83.55	99.77
		U-Net	Sentinel-2	56.00	85.15	84.11	97.27
			WorldFloods	73.86	79.99	78.36	98.91

† Permanent water obtained from the yearly water classification product of Pekel et al.[?] available at the Google Earth Engine[?].

Table 4. Recall and IoU of the water class for each of the flood events in the test dataset.
